# Role of phasiRNAs from two distinct phasing frames of *GhMYB2* loci in *cis*- gene regulation in the cotton genome

**DOI:** 10.1186/s12870-020-02430-3

**Published:** 2020-05-15

**Authors:** Ting Zhao, Xiaoyuan Tao, Menglin Li, Mengtao Gao, Jiedan Chen, Na Zhou, Gaofu Mei, Lei Fang, Linyun Ding, Baoliang Zhou, Tianzhen Zhang, Xueying Guan

**Affiliations:** 1 College of Agriculture and Biotechnology, Zhejiang University, Hangzhou, Zhejiang China; 2 State Key Laboratory of Crop Genetics and Germplasm Enhancement, Cotton Hybrid R & D Engineering Center (the Ministry of Education), College of Agriculture, Nanjing Agricultural University, Nanjing, Jiangsu China

**Keywords:** Cotton, Fiber, phasiRNAs, RLM-RACE

## Abstract

**Background:**

Phased small interfering RNA (phasiRNA) is primarily derived from the 22-nt miRNA targeting loci. *GhMYB2*, a gene with potential roles in cotton fiber cell fate determination, is a target gene of miR828 and miR858 in the generation of phasiRNAs.

**Results:**

In the presented work, through the evaluation of phasing scores and phasiRNA distribution pattern, we found that phasiRNAs from *GhMYB2* were derived from the 3′ cleavage fragments of 22-nt miR828 and 21-nt miR858 respectively. These two miRNA targeting sites initiated two phasing frames on transcripts of one locus. By means of RNA ligase-mediated rapid amplification of cDNA ends (RLM-RACE), we further demonstrated that phasiRNAs derived from the two phasing frames played a role in *cis*-regulation of *GhMYB2*. The phasiRNAs derived from *GhMYB2* were expressed in the somatic tissues, especially in anther and hypocotyl. We further employed our previous small RNA sequencing data as well as the degradome data of cotton fiber bearing ovules, anthers, hypocotyls and embryogenic calli tissues published in public databases, to validate the expression, phasing pattern and functions of phasiRNAs.

**Conclusions:**

The presenting research provide insights of the molecular mechanism of phasiRNAs in regulation of *GhMYB2* loci.

## Background

The phased small interfering RNAs (phasiRNAs) exist in a wide variety of plant genomes, from algae and moss species to monocot and dicot species [1]. The first reported phasiRNA was trans-acting small interfering RNA (tasiRNA) derived from long non-coding RNA loci, namely *TAS* genes: *TAS1/2, TAS3* and *TAS4* [2]. As a specialized type of phasiRNAs, tasiRNAs originate from *TAS* gene transcripts and functionally act in *trans* to regulate mRNA at the post-transcriptional level [3]. The miRNAs that initiate phasiRNA generation are known as phasiRNA triggers [4]. *TAS1/2* is triggered by miR173 [2, 5, 6]. *TAS3* is triggered by miR390 [5, 7–9] and *TAS4* by miR828 [10–13]. In addition to triggering by a single miRNA, there is also an alternative pattern of phasiRNA generation, known as the two-hit model [7] and phasiRNA production can be triggered by two distinct small RNAs, as phasiRNA production can be triggered by two distinct small RNA targeting, as observed in the petunia genome [14].

Although the biogenesis of phasiRNAs has been explored in the past decade, our understanding of the expression and phasing pattern of phasiRNAs remains limited. The phasiRNA distribution studies have demonstrated their tissue specific expression pattern. For example, phasiRNAs is the predominant type of small RNA found in the anther of angiosperms [15]. Furthermore, analysis of the small RNA profiles in maize anther shows that phasiRNA expression varies during the cell development [16]. According to the small RNA length, phasiRNA pattern can be divided into 21-nt and 24-nt phasing intervals. 21-nt phasiRNAs are active prior to 24-nt phasiRNAs at various stages of the cell fate specification and differentiation while meiosis is associated with 24-nt phasiRNAs activation. The latest DNA modification assays show that CG, CHG and CHH methylation levels are high in the phasiRNAs loci for both 21-nt and 24-nt groups in meiocytes [15]. These features suggest that phasiRNAs play an important role in anther development and potentially in epigenetic modification as well.

Recent studies on phasiRNAs in plant suggest that phasiRNAs function as small interfering RNA (siRNA) in critical agronomic trait controlling. For example, miR2118 is expressed in the panicle development stages in rice [17] and other seed plant species [18]. Further forward genetics and transgenic evidences demonstrate that miR2118 triggered *Pms1* locus to generate phasiRNAs in determining photoperiod-sensitive male sterility (PSMS) in rice [19]. Selective studies have shown that miR828 targets are involved in abiotic stress responses in *Arabidopsis* [13], apple [20], and sweet potato [21], as well as in flavonoid biogenesis [22, 23] and epidermal fiber cell development in cotton [24].

The functional pattern of phasiRNA include the cleaving of the target gene sequence according to the reverse complementary manner. Some *TAS2*-derived siRNAs can recognize pentatricopeptide repeat (PPR) protein coding genes in this way [25]. A recent functional study reports that the rice AGO5 homolog, MEL1, preferentially binds to phasiRNAs [26]. This phenomenon was highly associated with the miR2118-targeted sequences and miR2118-triggered phasiRNAs.

PhasiRNAs in cotton genome remains largely unknown. Eleven genes in Upland cotton were predicted to be *TAS* genes [27]. Moreover, some 200 phasing loci have been identified in sea-island cotton [28]. Previously, we made the first report of a functional phasiRNA-deriving gene in the cotton genome, *GhMYB2*, targeted by miR828 and miR858 [24]. *GhMYB2* encodes a MYB transcriptional factor that plays a role in plant epidermal cell fate determination. Promoter activity assay and mRNA in situ data demonstrate that *GhMYB2* expression predominantly occurs in cotton seed fiber cells during the cell differentiation stage [29, 30]. The impact of miR858 on the phasiRNA biogenesis derived from *GhMYB2* remains unknown. The function of the *GhMYB2*-generated phasiRNAs are not clear, yet. Here we examined both small RNA sequencing data from our previous studies of cotton leaf and ovule tissue, as well as mRNA degradome data on cotton fiber-bearing ovules, anthers, hypocotyls and embryogenic calli (EC), which we obtained from an online published database. Reviewing these sources in conjunction, we have further explored the new phasing pattern driven by two microRNA-targeting sites on *GhMYB2* and the *cis*-regulatory role of *GhMYB2*-derived phasiRNAs during the early stage of cotton fiber differentiation.

## Results

### Cotton *GhMYB2* generates phasiRNAs in two phases

**Fig. 1:**
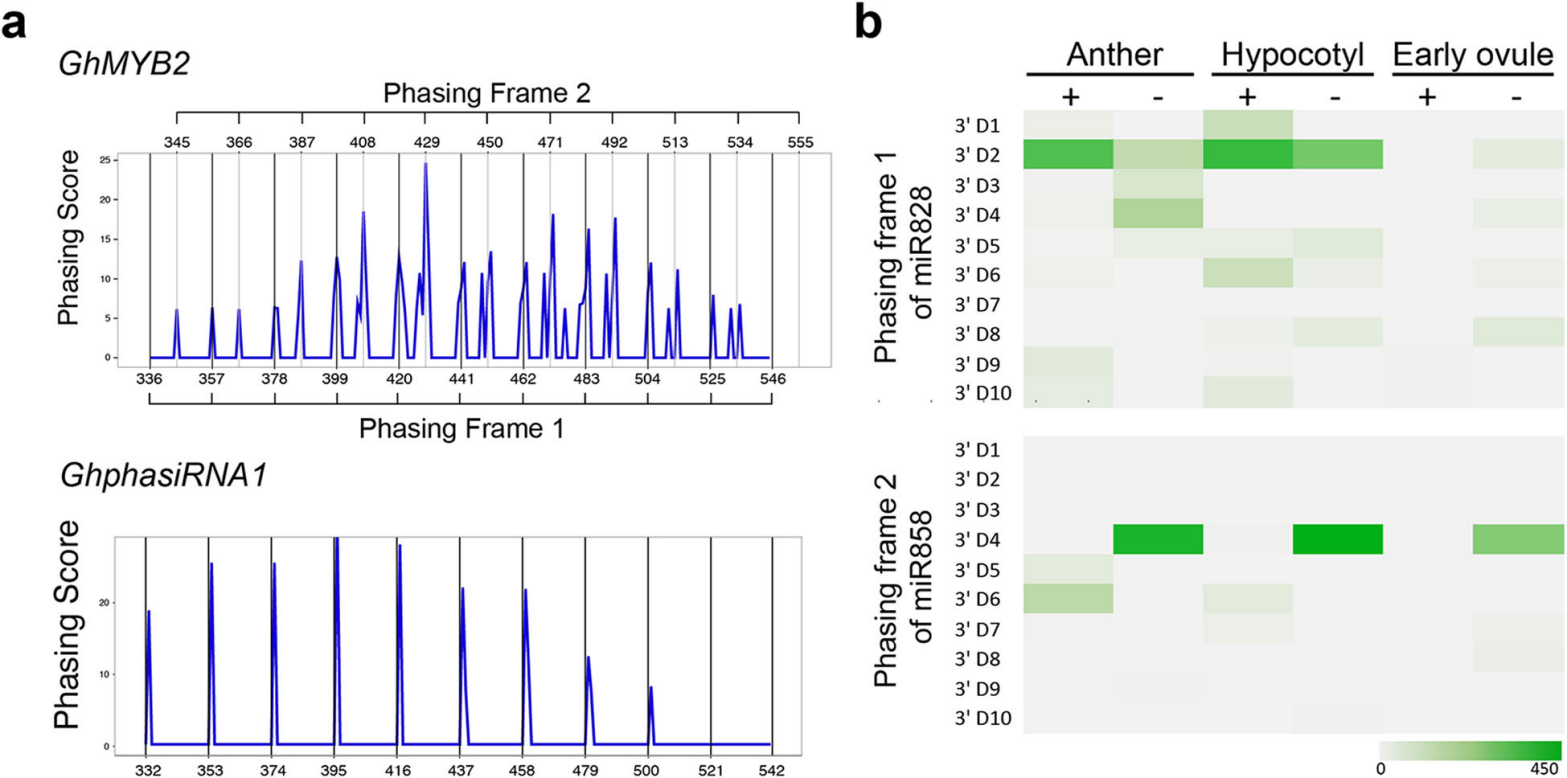
Alternative phasing of phasiRNAs on the *GhMYB2* loci in the cotton genome. **a**, Phasing score curve (see method) showing Phasing frame 1 (PF1) and Phasing Frame 2 (PF2) at the 21-nt interval on the *GhMYB2* loci. FR1 confirmed the phasing changes starting from miR828 cleavage site. **b**, Heat map showing the phasiRNA density over the 2 phasing frames in anther, hypocotyl and early ovule tissues. The sense and antisense strands are indicated by “+” and “-”, respectively

PhasiRNA-deriving genes undergo a phase-change after the formation of dsRNA to generate siRNA in-phase, as reported in the model plant, *Arabidopsis* [31]. To examine whether *GhMYB2* can derive siRNA in-phase, we adapted the calculation of phase score to evaluate the level of phasiRNAs of *GhMYB2* using the small RNA library of ovule tissue form upland cotton (*Gossypium hirsutum*, accession Texas Marker-1, TM-1) harvested on 0 DPA (day post anthesis). As shown in Fig. 1a, the phasing curve patterns of *GhMYB2* presented clear peaks in phase of 3′ end by miR828 cleavage site. This confirmed our previous reports on *GhMYB2* derived phasiRNAs triggered by miR828 [24]. Among the phasiRNA-deriving gene candidates, *Gh_D07G1901* (miR390 targeting) was predicted to be equivalent to *GhphasiRNA1* in the cotton genome. The phasing curve on *GhMYB2* was not as regular as those of *GhphasiRNA1*. Although the 21-nt phasiRNA interval was clearly observed, alternative peaks were also present in ovules and other tissues. Only the action of *GhphasiRNA1* in hypocotyls generated a smooth phasing curve at the 21-nt interval (Fig. 1a). We examined the pattern of the alternative peaks by analyzing their intervals. The 21-nt intervals were observed in the curve (Fig. 1a). Further examination of phasiRNA abundance and location indicated that the peaks of the *GhMYB2* phasing curve were derived from the 3′ cleavage site of the miRNA858 target (Fig. 1b, Supplemental table 1). Moreover, minor peaks were also observed repeatedly in the ovule and anther tissues. Therefore, we confirmed that *GhMYB2* can generate siRNA in phase on the 3′ cleavage fragment of both miR828 and miR858. Correspondingly, we named the phasing frame from the 3′ cleavage fragment of miR828 Phasing frame 1 (PF1). The alternative phasing frame from miR858 was named Phasing frame 2 (PF2). More phasiRNAs were found in phase of PF2 than PF1, as shown in Fig. 1a and b.

In contrast to the alternative phasing in the two-hit model triggered by miR390 [7], the distance between miR828 and miR858 on *GhMYB2* was as short as 12 bp. There was insufficient phasing space (21 nt for one small RNA in phase) between these two miRNA targeting sites, given that most of the phasiRNA triggers are 22-nt miRNAs. Due to the observation of phasiRNAs from two phasing frames, the phasing process should initiate from the miR858 and miR828 cleavage fragments respectively on independent transcripts. Given that miR858 itself cannot trigger phasiRNA genesis, the phasing process initiated on miR858 cleavage site on endogenous *GhMYB2* might be affected by miR828 triggered phasing change. The two miRNA sites triggered the phasiRNA genesis from two miRNA 3′ cleavage fragments should be on distinct starting site.

### *GhMYB2* -derived phasiRNA can degrade the *GhMYB2* by *cis-* effect

**Fig. 2:**
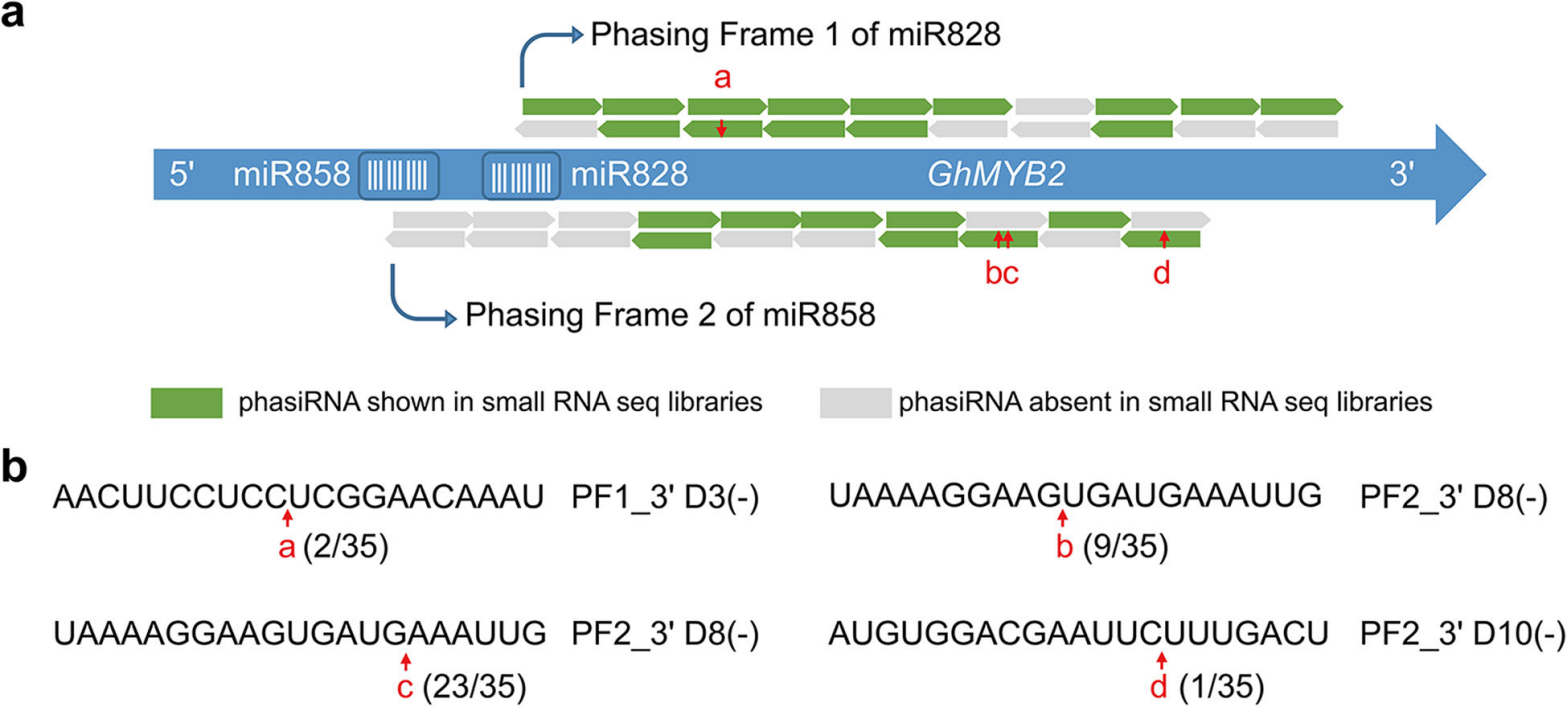
Cis-effects of phasiRNA derived from *GhMYB2* RNA in anther, hypocotyl and EC tissues. **a**, Schematic phasing model for the hypothesis that there is a phasing frame 1 (PF1) of the miR828 3′ cleavage site and phasing frame 2 (PF2) of the miR858 3′ cleavage site. The red arrows and letters represent the *GhMYB2* mRNA cleavage sites detected by RLM RACE. **b**, The sequences of each cleavage sites were enlarged. The frequency of each cleavage sites were shown. Out of 35 cleavage events detected, 2, 9, 23 and 1 events were on site a, b, c and d respectively. The detected cleavage sites were on PF1_3’D3(−), PF2_3’D8(−) and PF2_3’D10(−)

The two phasing frames of *GhMYB2* were visualized as the two major phasing peaks in the phasing score curve (Fig. 2a). These phasiRNAs might accelerate mRNAs degradation by forming dsRNA on the origin loci in *cis*. To examine this hypothesis, we detected the internal cleavage sites of *GhMYB2* mRNA by phasiRNAs. RNA ligase-mediated rapid amplification of cDNA ends (RLM-RACE) was performed using total RNA extracted from anthers in which the phasiRNAs were proactively expressed. Results indicated that *GhMYB2* was cleaved by phasiRNAs generated by both miR828 and miR858. The cleavage sites detected was exactly correspond to the phasiRNA predicted cleavage sites. Cleavage site a (indicated in Fig. 2b) was generated by the PF1-derived phasiRNA, PF1_3’ D3(−). The cleavage site was between 10th and 11th nucleotide of PF1_3’ D3(−). This pattern was similar with the miRNA-mediated mRNA slicing in plant genome. Similar pattern was observed on cleavage site b, which was generated by the PF2-derived phasiRNA, PF2_3’ D8(−). PF2_3’ D8(−) also generated another cleavage site c between the 15th and 16th nucleotide. Cleavage site d was on PF2-derived phasiRNA, PF2_3’ D10(−). Although this method is not for quantitative determination for cleavage products due to different efficiency in DNA fragment cloning, this results provided a direct evidence that *GhMYB2*-derived phasiRNAs can degrade the *GhMYB2* transcript in *cis*.

We further detected the degraded fragments of *GhMYB2* mRNA in anthers, hypocotyls and embryogenic calli (EC) (Supplemental table 2, seen in Methods). The number of *GhMYB2* mapped reads in the anther degradome was dramatically higher than in the hypocotyl and EC degradomes (Supplemental Fig. 2) (Wilcoxon rank sum tests, typically *P* < 0.01). *MYB109*, and GhphasiRNA1 did not follow this pattern (Supplemental Fig. 2). These data indicate that *GhMYB2* transcripts were preferentially degrades the by phasiRNAs of its own in *cis* in anthers.

### Tissue-specific expression of phasiRNAs at *GhMYB2* loci

**Fig. 3:**
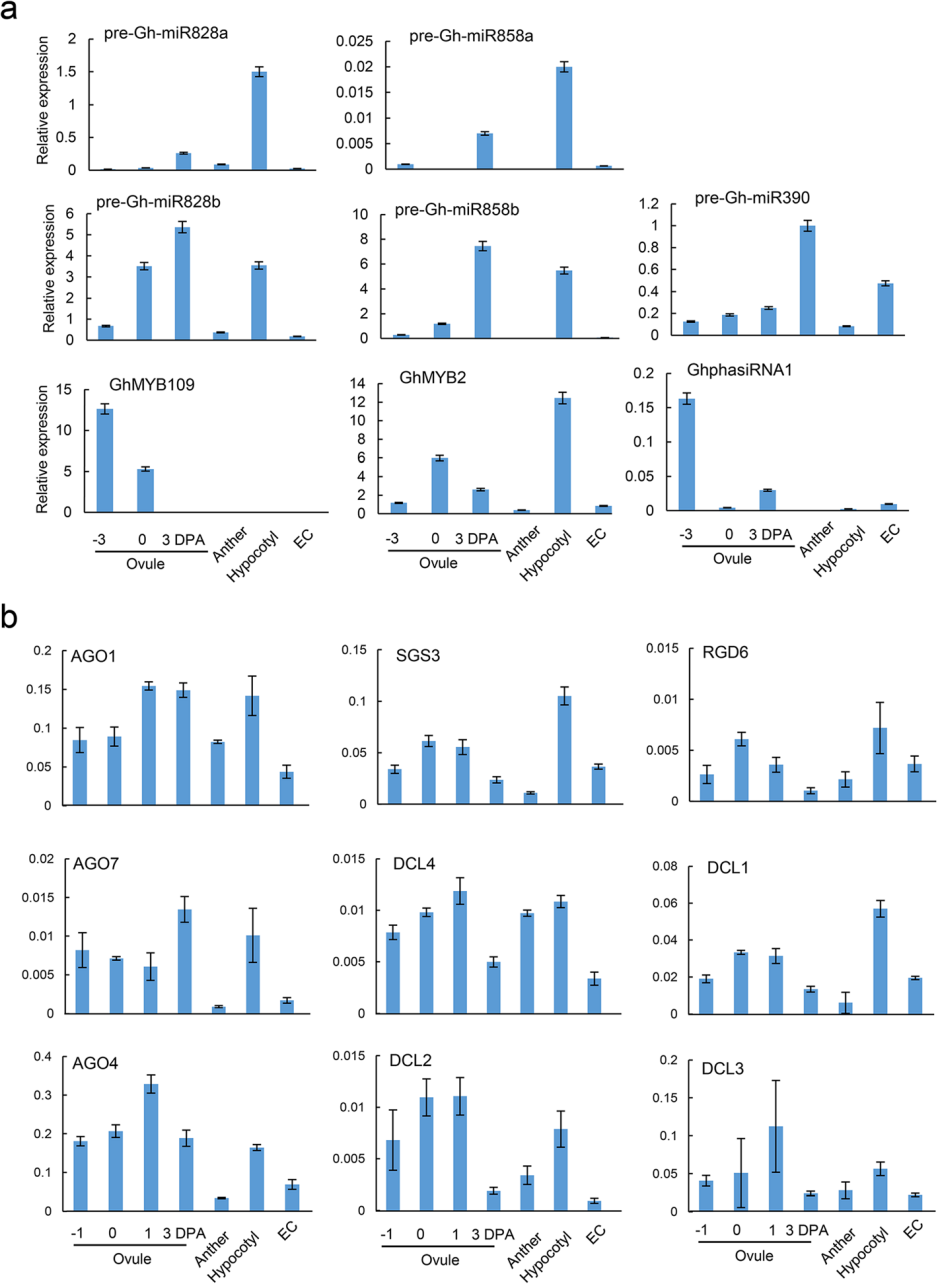
Expression pattern of phasing RNA triggering miRNA, their targeting genes and genes involved in tasiRNA biogenesis. **a**, qRT-PCR analysis of precursor forms of miRNAs including GhmiR828, GhmiR858 and their targeting gene *GhMYB2*. GhmiR390 and its targeting gene GhphasiRNA1 was served as the positive controls for phasiRNA generation. *GhMYB109* was miR828 targeting gene and used as negative control for phasiRNA deriving. **b**, qRT-PCR analysis of genes involved in tasiRNA biogenesis, including *AGO1*, *SGS3*, *RDR6*, *DCL4*. The genes of *AGO4*, *AGO7*, *DCL1*, *DCL2* and *DCL3* were used as internal controls for gene redundancy in early ovules from − 1, 0, 1 and 3 days post anthesis (DPA), anthers, hypocotyls and seeds. Three biological replicates were used for each tissue

In order to determine whether phasiRNA generation is associated with the expression of the origin gene, we examined the expression of the two miRNA precursors and *GhMYB2* in cotton ovules, anthers, hypocotyls, and EC tissue using qRT-PCR. Results indicated that, the expression levels of GhmiR828 and GhmiR858 precursors were high in ovules at 0 and 3 days post anthesis (DPA), hypocotyls, but low in early ovules at − 3 DPA, anthers and EC tissues (Fig. 3a, Supplemental table 6 and Supplemental fig. 3). The *GhMYB2* expression level was highest in hypocotyl tissues and lowest in early ovules of − 3 DPA, anthers and EC tissues, with levels in early ovules of 0 and 3 DPA in between (Fig. 3a). The abundant accumulation of miR828 and miR858 at ovules of 0 DPA and 3 DPA was consistent with previous discovery [24]. Notably, the 3 DPA ovules with relatively higher miR828 and miR858 levels when compare with 0 DPA ovules, showed relatively lower *GhMYB2* mRNA expression, indicating the precise regulation of *GhMYB2* genes by miRNA during cotton fibre development.

### The molecular basis of tissue-specific expression of *GhMYB2* derived phasiRNAs

The cotton genome underwent multiple rounds of whole genome duplication. We speculate that cotton genome carries redundant proteins in phasiRNA biogenesis. To test this hypothesis, we examined the endogenous expression of *AGO1, SGS3, RDR6* and *DCL4* in cotton ovules, anthers, hypocotyls, and EC tissue using both mRNA sequencing data and qRT-PCR. We discovered that genes responsible for phasiRNA biogenesis were actively expressed in early ovule tissues, which corresponds to small RNA activity in fertilized ovules (Fig. 3). The expression of these genes was lower in anther tissues. *AGO1* expression was comparable in ovule and hypocotyl tissues, but *RDR6* and *DCL4* expression was significantly higher in hypocotyls than in other tissues (Fig. 3b). All of these gene expression patterns agree with the high abundance of phasiRNAs derived from *GhMYB2* in hypocotyls (Fig. 3a). While the absence of phasiRNA biogenesis in EC tissue corresponded to the undetectable levels of GhphasiRNA1 and *GhMYB2* phasiRNAs in undifferentiated EC tissues. In the reproductive tissues, *AGO1* expression was very active (Fig. 3b), a pattern similar to that observed in rice [32], which we noted earlier. The expression of *DCL* genes was also higher in hypocotyl tissue than in anther tissue (Fig. 3b). We know that in *Arabidopsis,* the function of *DCL4* can be carried out by *DCL1*. But unlike the 21-nt final products of *DCL4*, *DCL1* generates more 22-nt siRNA [33] . In this same way, GhphasiRNA1*,* and *GhMYB2* in the cotton genome generated not only 21-nt phasiRNAs, but they can also generate 18–29 nt siRNAs (Supplemental dataset 1). To unveil the potential redundant functions of phasiRNA-biogenesis proteins in cotton, we extended the corresponding homologs in genome wide. The expression patterns of *AGO, RDR6, SGS* and *DCL* homologs were extensively examined via mRNA-seq analysis in a selection of 14 cotton tissues. These genes showed a distinct tissue specific pattern in general which was similar to the pattern shown with the qRT-PCR analysis (Supplemental fig. 2, Supplemental table 6). These results excluded the potential error introduced by the internal control gene selection of qRT-PCR. Therefore, AGO1 might lead the slicing of miRNA/mRNA, but DCL1 and DCL4 likely carry out the final cleavage of dsRNA. The proactive expression activity of Argonaute and DCL coding genes in somatic tissues than undifferentiated cell might be responsible for the abundant expression of phasiRNAs.

### *GhMYB2* loci in the cotton genome generate phasiRNAs in somatic tissues, but not in undifferentiated cells

**Fig. 4:**
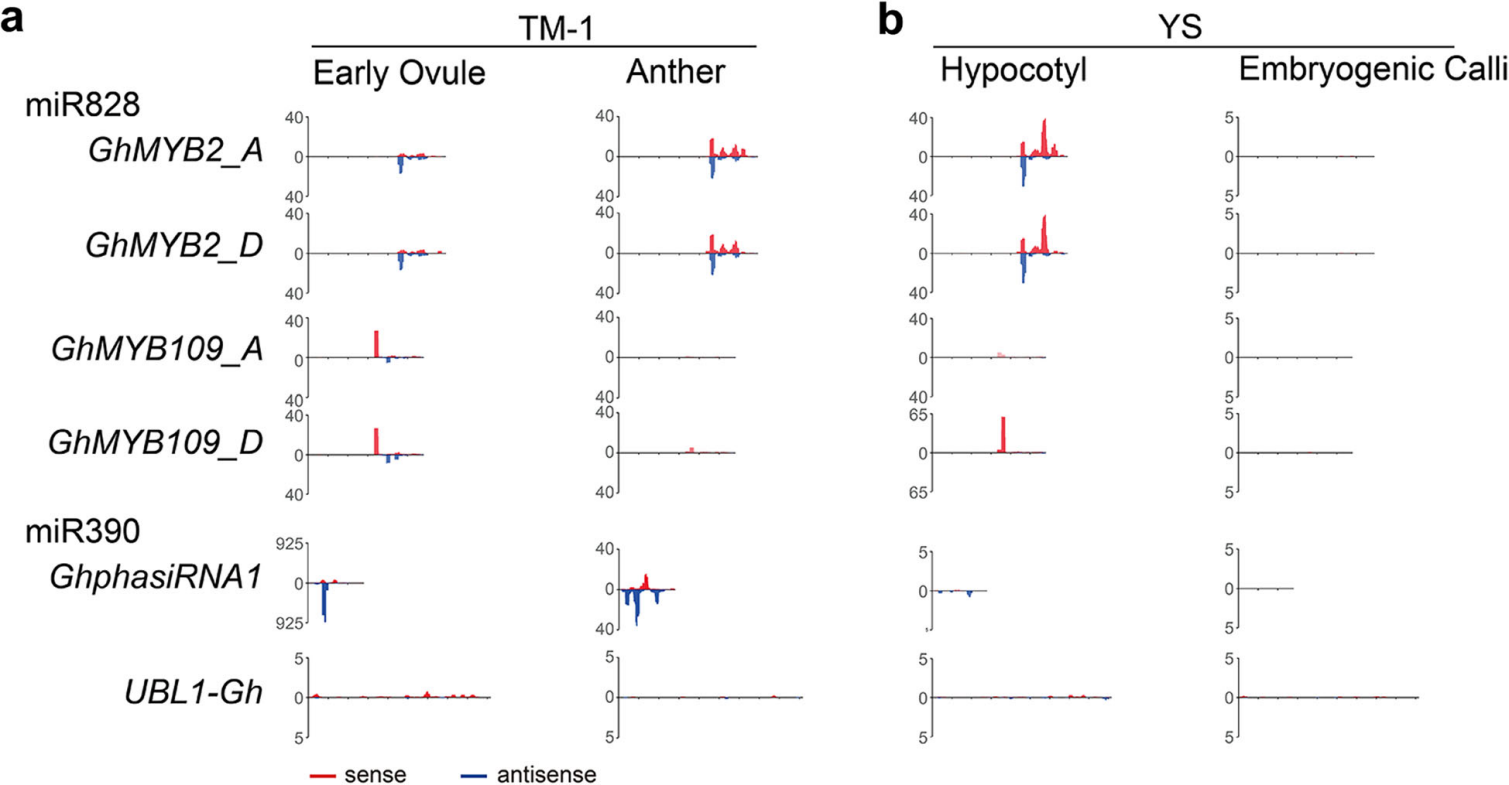
*GhMYB2* generated phasiRNAs in different cotton tissues. Small RNA mapping results showing the tasiRNA distribution on the *GhMYB2* locus in early ovule and anther from accession TM-1 (**a**), hypocotyl and embryogenic callus (EC) tissues from accession YS (**b**). Small RNA mapping comparisons were normalized by the counts per million reads (CPM) from each tissue sample. Both A and D subgenome allele for *GhMYB2* and *GhMYB109* were presented. Due to the very few polymorphism, small RNA mapping densities did not exhibit distinctive difference between either allele. *GhMYB109* was the negative control for the miR828 targeting. *Gh_D07G1901* (miR390 targeting) were predicted to be equivalent to GhphasiRNA1 in the cotton genome and therefore served as the positive controls for phasiRNA deriving genes. *UBL1-Gh* was the negative control for non-miRNA targeting loci

*GhMYB2* is a coding gene known to generate phasiRNAs in the fiber-bearing tissues of cotton [24]. Both the phasiRNA trigger, miR858 and miR828 and the *GhMYB2* gene expression showed tissue-specific pattern is consistent with the GhMYB2 function in fiber cell regulation. In order to detect *GhMYB2* loci capability of generating phasiRNAs in a variety of tissues, we investigated potential phasiRNA-deriving genes in the cotton genome. Our previous studies predicted that *GhMYB109* is targeted by miR828 to generate phasiRNAs. However, small RNA mapping and miRNA cleaving test has suggested that *GhMYB109* is not in fact a phasiRNA-deriving gene in cotton. Consequently, in this study *GhMYB109* was employed as a negative control for phasiRNA generation. We used the reverse complementary rule of miRNA targeting to predict the target sites of miR173 and miR390 in the Upland cotton genome (*G. hirsutum,* TM-1), allowing three mis-matches and one T-G bulge. We then identified 45 of the predicted miR173 targets and 13 of the predicted miR390 targets as potential phasiRNA-deriving loci in the cotton genome (Supplemental table 3). *GhphasiRNA1* presented a particular abundance of small RNA transcripts in early ovule, anther, hypocotyl and EC (Fig. 4). Therefore, they were selected as positive controls for phasiRNA-deriving cotton genes. Finally, the cotton *GhUBL1* gene, which encodes ubiquitin, was selected as the non-miRNA targeting control.

Small RNA density plot used the perfect mapped reads from each sample. To compensate the lacking of biological replication from each tissues sample, we selected three somatic tissues, early ovule, anther and hypocotyl as independent biological samples compared with the undifferentiated sample, EC tissue (Supplemental table 2).

We observed that *GhMYB2* loci from both the A and D subgenomes of cotton generated high densities of small RNA distribution in the ovule, anther and hypocotyl (Fig. 4), similar to *GhphasiRNA1*. Because the low polymorphism between the A and D alleles, *GhMYB2* small RNA density didn’t exhibit obvious differences allelically (Fig. 4a). As expected, *GhMYB109*, in a pattern similar to that of *GhUBL1*, did not generate high levels of small RNA in any tissues (Fig. 4). In addition, we noted that neither *GhMYB2* nor *GhphasiRNA1* generated much siRNA in the embryogenic callus (EC) tissue, as shown in Fig. 4b. These results suggest that *GhMYB2* can express and produce phasiRNAs in differentiated somatic cells but not in undifferentiated EC cells due to the tissue-specific expression pattern.

### *GhMYB2*-derived phasiRNAs take potential roles in *trans*- regulate downstream targets

**Fig. 5:**
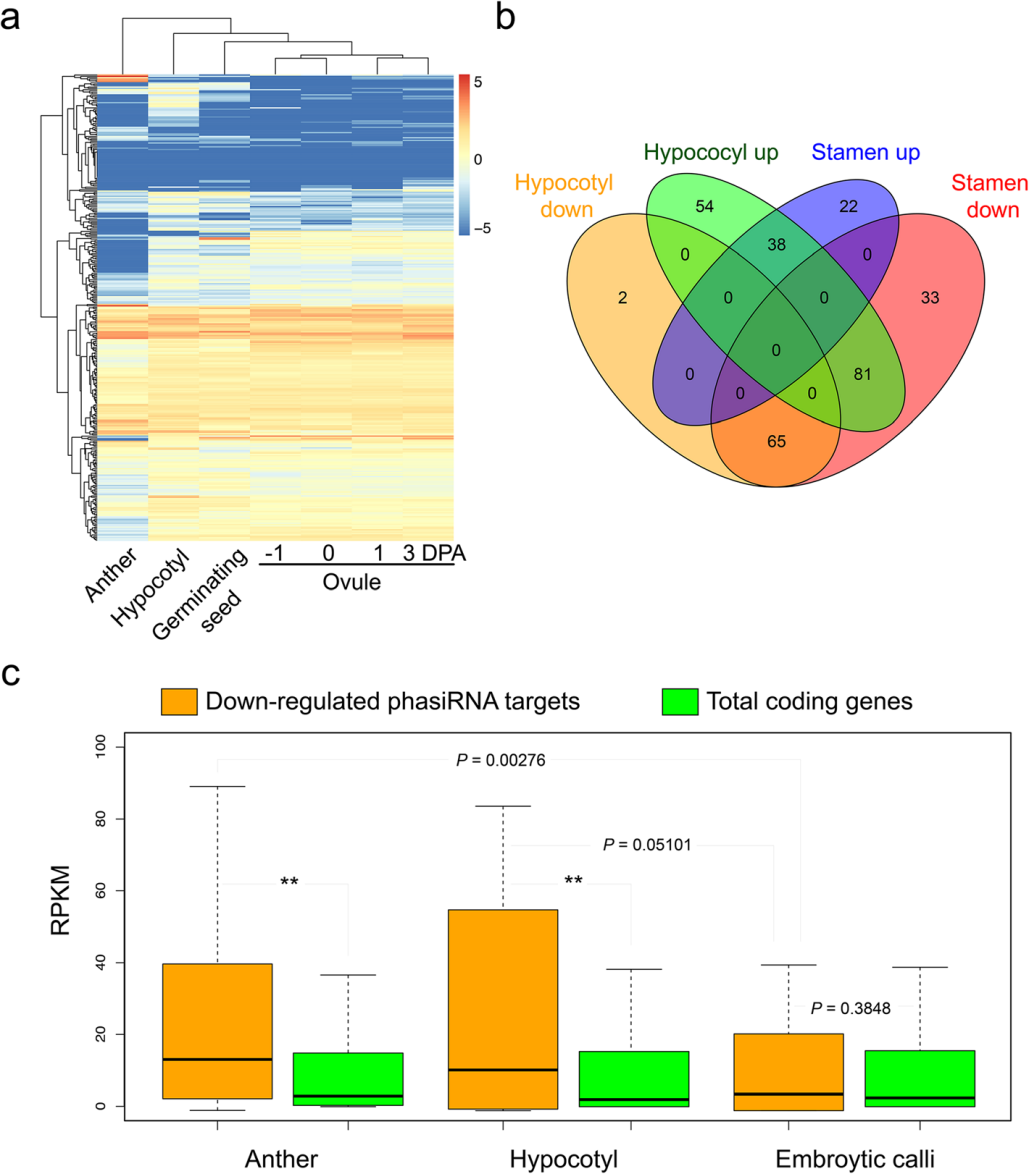
phasiRNA target gene expression profile. **a**: Heat map displaying the expression pattern of the predicted phasiRNA targets in the cotton genome. PhasiRNAs were derived from *GhMYB2* loci. Only conserved tasiRNAs that were consistently expressed in ovule, anther, hypocotyl tissues were selected. The phasiRNA targets were clustered according to their tissue expression patterns. **b**: Venn diagram illustrating differences between the expression patterns of the predicted phasiRNA target genes. All comparisons were against to the early ovule tissues. **c**, Degradome analysis of the down-regulated phasiRNA targets in anther, hypocotyl and EC tissues. Box plot showing the abundance of degraded fragments in anther, hypocotyl and EC tissues using RPKM values. The central line for each box plot indicates the median. The yellow represents the population of down-regulated phasiRNA targets in anther and hypocotyls tissue. The total levels of degraded fragments of coding genes were used as a background control (green). The *P* value was calculated using the Wilcoxon rank sum test

To further examine the potential functions of *GhMYB2*-derived phasiRNAs, we predicted the phasiRNA targets in the cotton genome according to the reverse complementary rule, allowing 2 mismatches (Supplemental dataset 2). To avoid the effects of random small RNA, only the overlapping phasiRNAs from ovules, anthers and hypocotyls were selected for the target predicting investigation. The expression of phasiRNA targets in cotton ovules, anthers and hypocotyl tissue are presented in the heatmap in Fig. 5a. The differentially expressed genes (DEGs) of phasiRNA target genes in anthers and hypocotyls are shown in a VENN diagram in Fig. 5b. Only 41.3% (38/92) and 63.3% (38/60) of all target genes were common, up-regulated genes in hypocotyls and anthers, respectively. However, 97.2% (65/67) of down-regulated target genes in hypocotyls overlapped with those in anthers. The down regulated target genes in anthers were significantly enriched in hypocotyls (Fisher’s exact test, *P* < 6.54e^− 15^). The high proportion of common, down-regulated genes in anthers and hypocotyls suggests that the phasiRNAs in these tissues target similar genes in similar functional pathways. GO enrichment analysis indicated that these phasiRNA targets were involved in cytoskeletal anchoring at plasma membrane at high Rich Factor (Supplemental Fig. 4). The cell skeleton arrangement is related to the structure of cell shape. We found that the enrichment of down-regulating genes in this category reflects the unique function of *GhMYB2* as a phasiRNA-deriving gene in specific cell morphology, especially in fiber cells (Supplemental table 4).

Anthers were highly enriched with 21- and 24-nt phasiRNAs [16]. In order to examine whether the suppression of phasiRNAs target expression in anthers and hypocotyls is due to *GhMYB2*-derived phasiRNA, we compared the degraded fragments of the 65 phasiRNA targets to the total genome. To avoid the effects of random degraded fragments, the reads mapped on 5′ end of the phasiRNAs targeting sites were filtered out. The median level of degradation of the 65 phasiRNA targets in anthers and hypocotyls was twice that of the total genome (Fig. 5c) (Wilcoxon rank sum tests, anther vs EC *P* = 0.0276; hypocotyl vs EC *P* = 0.05101). The phasiRNA targets degraded fragments were significantly higher than the total coding gene background in anther, hypocotyl and EC tissues (Wilcoxon rank sum tests, typically *P* < 2.2e-^16^). Therefore, the differences in expression of the 65 phasiRNA target genes might be caused by phasiRNA *trans*- regulation. This suggested that the phasiRNAs generated by *GhMBY2* in cotton could down-regulate target gene expression by increasing the degradation of their mRNA.

## Discussion

### *GhMYB2* has two phasing frames for phasiRNAs production in the cotton genome

PhasiRNAs is a unique type of small RNA. It can be derived from trigger miRNAs with a length of 21 nt or 22 nt [34]. In the past decade, several phasiRNA triggers have been identified in moss [35], olives [36], chinese fir [37], apple [20], rice [38], cotton [27, 39], sorghum [40], *Dimocarpus longan Lour* [41] and soybean [42]. One model of phasiRNA triggering consists of a ‘one-hit’ system that usually involves a 22-nt microRNA triggers. MiR173 is such conserved phasiRNA trigger in plant genomes. Another model of phasiRNA biogenesis consists of a ‘two-hit’ system, which uses two 21-nt microRNAs per mRNAs transcript [34]. Representative *TAS* genes of ‘two-hit’ model carry two miR390 targeting sites found in moss and *Arabidopsis* [7]. These mRNAs carry two miR390 reverse complementary sites across a 200–300 nt region. PhasiRNAs are found to be active in the region between the two miRNA targeting sites in phase of the 5′ to the cleavage site of miR390. Alternative phasing in this region has also been reported [7]. There are two hypotheses for the cause of this alternative phasing: *cis*- activation of one of the phasiRNAs, or activation of the 5′ miR390 cleavage site.

Some of the miR828 targeting loci in apple and cotton genomes have been reported to be targeted by two miRNAs; miR828 and miR858 [20, 24]. Two-hit *GhMYB2* can generate phasiRNAs in phase of the 3′ miR828 cleavage site [20, 24]. In our present research, we observed that the *GhMYB2* loci actively generated phasiRNAs in the alternative phasing frame of the 3′ miR858 cleavage site and that *GhMYB2* can generate phasiRNAs on both PF1 and PF2 in multiple tissues.

Due to the existence of a double phasing frame for the loci, the phasing score curve for *GhMYB2* shows double peaks on each phase. This is not a typical phasing curve. Alternative phased small RNAs have never been considered in phasiRNAs analysis. Therefore, the conventional evaluation of phasiRNA prediction, which follows the principle of one phasing frame on one locus, could underestimate the amount of phasiRNAs in the genome. This revelation could in turn impact the analysis of the function of phasiRNAs.

### Tissue-specificity of phasiRNA expression in cotton

The abundance of phasiRNAs of origin loci vary from tissue to tissue. GhphasiRNA1 generates phasiRNAs in reproductive tissues (Fig. 4) and *GhMYB2* produces more phasiRNAs in vegetative tissues than in ovules (Fig. 4). It is likely, then, that the efficiency of phasiRNAs biogenesis differs between tissues.

The biogenesis of phasiRNAs requires a unique set of proteins, but collective data reveal the functional redundancy of the involved Argonaute and DCL protein families. In *Arabidopsis*, an Argonaute protein is thought to be required for the slicing of 22-nt miRNA-targeted mRNA [43]. In a recent report, those derived 21-nt phasiRNA was detected in an *ago1* null mutant [43]. The assimilated impacts of AGO2 and AGO7 in phasiRNA biogenesis [6, 8, 44] are redundant within the Argonaute protein family. Similarly, dicer-like proteins DCL1–4 digest dsRNA into small RNA [4] while several different DCL proteins carry out functions related to miRNA, siRNA and phasiRNAs. DCL4 specifically recognizes RDR6-generated double stranded RNA and cuts it into 21 nt RNA [2]. But in the absence of DCL4, this function is adopted by DCL1 with the product of phased small RNAs in length of 22 nt [33].

We observed the expression pattern of phasiRNA biogenesis related genes in cotton tissues. These results suggest that the phase-change of phasiRNAs loci is primarily dependent on the activity of Argonaute and Dicer-like proteins, which varies among tissues. The activity of *DCL1, DCL2, DCL3* and *DCL4* expression was consistently high in early ovule and fiber tissues (Fig. 3b). *RDR6* and *DCL4* mRNA expression pattern was identical in hypocotyls. *DCL1, DCL2* and *DCL3* were all found to be actively expressed in the hypocotyl (Fig. 3b). The expression of AGO1, AGO4 and AGO7 mRNA was also high in hypocotyls (Fig. 3b). Therefore, the redundant Argnaute and DCLs most likely slice the long dsRNA into small RNAs of different lengths and phases.

### The *cis*- function of phasiRNAs

PhasiRNAs is common in plant genomes, but little has been determined about the biological functions of these small RNAs. In 2014, Small RNA sequencing of maize anthers at different developmental stages led to the discovery that phasiRNA expression is associated with the meiosis progress [15, 16]. The expression of 21-nt phasiRNAs occurs prior to that of 24-nt phasiRNAs in anther development, which suggests that 21-nt phasiRNAs and 24-nt phasiRNAs play different roles in meiosis. Analysis of maize small RNA also revealed that phasiRNAs are the dominant small RNA population in anther tissue [16]. These findings began to elucidate the specific functions of 21-nt phasiRNAs. However, the precise function of these phasiRNAs has remained unknown.

**Fig. 6:**
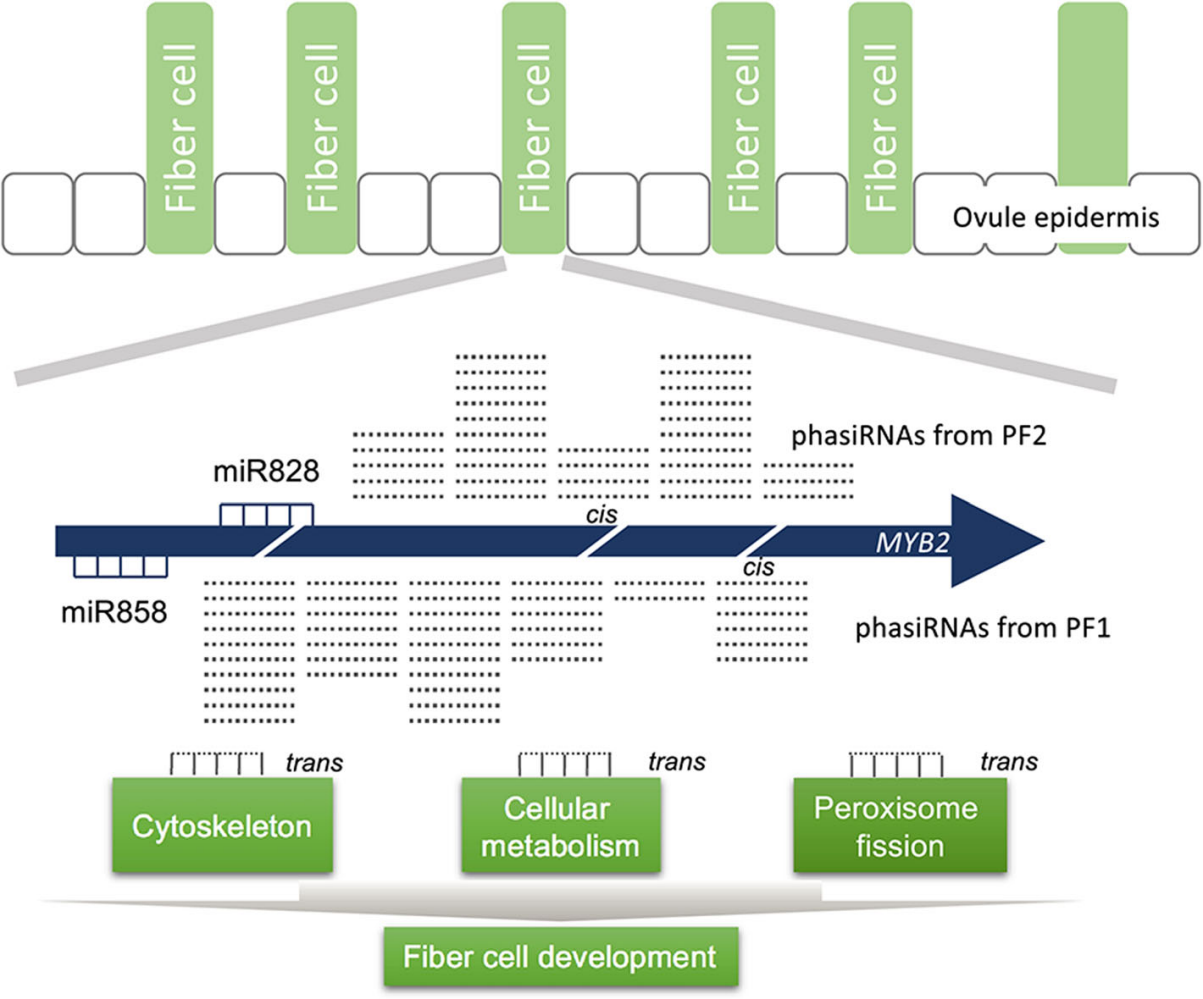
The working model of phasiRNA derived from *MYB2* locus in cis- and trans- regulation

In response, we tried to argue through RNA degradation data analysis that phasiRNA might play a role in mRNA degradation by *cis*-regulation (Fig. 6). The original phasiRNA location could be the primary target site of phasiRNAs, especially in anthers. Degraded fragments of *GhMYB2* mRNA are enriched in those tissues with high levels of phasiRNA generation. This phenomenon would seem to indicate that phasiRNA targets the original mRNA in a similar way to miRNA, in order to guide mRNA cleavage. However, we also found that the degraded fragments were not enriched in hypocotyl tissue, though phasiRNAs were expressed. Therefore, mRNA degradation is tissue-specific. This ultimately suggests that mRNA degradation must be under the control of other unknown elements in the genome beyond small RNA.

### The *trans*- effect of phasiRNAs on gene silencing

Small RNA plays unique roles in fiber development, and its process could be mirrored in phasiRNA function. The small RNA profiles of leaves and ovules have been found to be distinctly different in the early stages of fiber development and during and after fertilization [45]. Fuzz fiber differentiation is determined by the *N*_*1*_ gene, *GhMYB25-like/GhMML3* [46]; the suppression of fuzz is caused by small RNAs derived from the *GhMML3* locus. The small RNA from *GhMML3* in *N*_*1*_ is generated from dsRNA formed by the sense and antisense transcripts of *GhMML3*.

Although the biogenesis pathway is different, phasiRNAs could affect the genome in a way similar to small RNA from Natural antisense transcripts (NATs) [47]. Collective data suggest that NAT-derived small RNAs are involved in de novo DNA methylation and histone modification [46]. These small RNAs could follow the reverse complementary base pair rules to find the deductive target location with a relatively high accuracy. Herein we predicted the phasiRNA targets in the cotton genome, which appear to be suppressed in hypocotyls. The hypocotyl tissue was found to have the highest phasiRNA production from the *GhMYB2* locus out of all examined tissues. The suppression of these genes in the hypocotyl tissue suggests a correlation between phasiRNAs and its potential targets, which would mean that phasiRNAs could play a role in *trans*-gene regulation (Fig. 6).

## Conclusions

In this study, we have extensively analyzed the phasing pattern and function of *GhMYB2*-derived phasiRNAs. We integrated small RNA seq, mRNA seq and mRNA degradome data to investigate the behavior and functions of phasiRNA in the cotton genome. Using cotton *GhMYB2*, *TAS1/2*, *TAS3* as research examples, we found that *GhMYB2* can generate phasiRNAs on two phasing frames; one at the 3′ cleaved end of miR828 and one at the miR858 target site. The known two-hit trigger of TAS by two miR390 sites only presents one phasing frame. This one-phasing frame principle for phasiRNA biogenesis inspired a huge amount of genome-wide phasiRNA examination in *Arabidopsis*, rice, maize and many other species. Our novel finding of an alternative phasing frame on one locus may modify the formula for phasiRNA prediction and evaluation. PhasiRNAs can *cis*- regulate *GhMYB2* mRNA by degrading it. In our study, we provide the direct evidence of the endogenous functions of phasiRNA in a plant genome.

## Methods

### Cotton material growth conditions and cotton tissue collection

Upland cotton (*Gossypium hirsutum*) standard accession Texas Marker-1 (TM-1) was obtained from Crop Research Division, ARS, USDA, College Station, Texas. TM-1 was grown in the greenhouse with a 28 °C, 16/8 h (light/dark) light cycle. Ovule-bearing fibers on the epidermis and anther were harvested on the day of flowering (0 days-post-anthesis, DPA) for small RNA sequencing sampling. Hypocotyl tissues were harvested from 4-week old cotton seedlings. Embryogenic calli tissues were harvested from calli grown from hypocotyls tissues in embryogenic media for 8 weeks. At least three biological replications were used for each tissue. Ovules, hypocotyl, anther and embryogenic calli tissues were harvested for RNA extraction and qRT-PCR validation.

### Small RNA sequencing, degradome and cotton mRNA sequencing data sources

Small RNA sequencing data for cotton ovules from *Gossypium hirsutum*, acc. TM-1 were previously produced in our lab and published in The National Center for Biotechnology Information, Gene Expression Omnibus (NCBI GEO, https://www.ncbi.nlm.nih.gov/geo/) with accession number of PRJNA293171, TM-1 small RNA accession SRX1174194. The RNAs from − 1, 0, 1, 3 and 5 DPA ovule tissues were mixed to prepare the small RNA library of early ovule. Small RNA sequencing data for *G. hirsutum* anthers*,* hypocotyls and embryogenic calli were downloaded from NCBI GEO, accession numbers GSE43531 (anther, small RNA and degradome) and GSE41132 (hypocotyl and embryogenic calli, small RNA and degradome) [48]. The reference genome was the Upland cotton TM-1 genome [49].

### RNA extraction and real-time quantitative reverse transcription PCR (qRT-PCR)

RNA extraction was conducted using the Plant RNA Express Extraction kit (RK2002, Zoonbio Biotechnology) and RNA quality was examined by Nanodrop. Reverse transcription was conducted using the first strand cDNA synthesis kit (PC24-50 T, Zoonbio Biotechnology) and qRT-PCR was performed on an ABI 7500. The relative gene expression was normalized to the cotton *Histone 3* gene [46] using the ΔΔCt method [50]. The qRT-PCR primer set was designed as described [51]. All gene accession number and qRT-PCR primer information are listed in supplemental table 5. MiRNA precursors were listed in supplemental table 6.

### Bioinformatics analysis of small RNA mapping and degradome mapping

Raw reads of small RNA and degradome sequencing libraries were obtained from NCBI GEO published database, representing three type of somatic tissues of ovule, anther and hypocotyl, and one undifferentiated tissue, embryogenic calli. Because lacking of plenty biological replicates for each sample, three different somatic tissues were employed as independent biological samples for comparison. After adapter clipping, the clean reads from each library were mapped to the candidate genes using Bowtie [52] (settings -a -v 0), allowing no mismatches and retaining all alignments. The retained sRNA reads were 14,956,687 for early ovule, 16,347,976 for anther, 24,948,581 for hypocotyl and 25,984,975 for EC (Supplemental table 2). The small RNA comparisons among tissue samples were normalized by counts per million (CPM) value on the same phasiRNA deriving locus. The small RNA total mapping rate was 90–93% (Supplemental table 2). The unique mapping rate and multi-mapping rate were all above 40% for each library (Supplemental table 2), representing a fine small RNA profile for further study. The small RNA densities were normalized by counts per million (CPM) value.

The degradome qualifications were examined with Ylab: RPKM. The central line for each box plot indicates the median. The top and bottom edges of the box indicate the 25th and 75th percentiles and the whiskers extend 1.5 times the interquartile range beyond the edges of the box. Significance was evaluated using Wilcoxon rank sum tests.

### Mapping of the *MYB2* mRNA internal cleavage sites by phasiRNAs

For mapping the internal cleavage site in *MYB2* mRNA, RNA ligase-mediated rapid amplification of cDNA ends (RLM-RACE) was done using the FirstChoice® RLM-RACE Kit (Invitrogen). Total RNA was isolated from the tissues used as indicated in the results, include anther, hypocotyl, ovules and EC. Total RNA (4 μg) was directly ligated to the GeneRacer RNA Oligo adapter without the treatment of calf intestine alkaline phosphatase (CIP) and tobacco acid pyrophosphatase (TAP). Outer 5′ RLM-RACE PCR was performed using the 5′ RACE Outer Primer (5′-GCTGATGGCGATGAATGAACACTG-3′) and 5’RACE gene specific outer primer (5′-TACCATTGCTAATGGATCCTGTTGAT-3′) to prime first strand cDNA synthesis in reverse transcription reaction. Inner 5′ RLM-RACE PCR was then applied using 5’RACE inner primer (5′-CGCGGATCCGAACACTGCGTTTGCTGGCTTTGATG-3′) and 5’RACE gene specific innner primer (5′-CATCAAGTTCAAGGAACTTATTCACC-3′). PCR products were gel purified and cloned into pGEM-T Easy vector (Promega, Madison, WI) for sequencing.

### Phase transformation evaluation

Phase transformation data were produced using the following optimized equation [31]:

$$ P=\ln \left[{\left(1+\sum \limits_{i=1}^{10} ki\right)}^{n-2}\right],P>0. $$

where n is number of phase cycle positions occupied by at least one small RNA read within an eight-cycle window, and k is the total number of reads for all small RNAs with consolidated sequence in a given phase within an eight-cycle window. The cutoff of the phasing score was arbitrarily set at 5.

### PhasiRNA target prediction

Based on the phasiRNAs from *GhMYB2A* and *GhMYB2D* in all ovules, anthers, hypocotyls and embryogenic calli as the conserved phasiRNA query, the phasiRNA targets in the Upland cotton TM-1 genome were predicted [49]. The method of phasiRNA target prediction was that of the TargetFinder (https://github.com/carringtonlab/TargetFinder) online tools. The parameters were set to a TargetFinder score of ≥140 and a small RNA::mRNA interaction secondary structure maximum free energy (MFE) of ≤ − 20 kcal/mol.

## Supplementary information

**Additional file 1: Figure S1.** The secondary structure of miR828 and miR858 precursors

**Additional file 2: Figure S2.** The heatmap of mRNA activity showing the AGO, DCL, RDR6, SGS3 homologs in upland cotton genome based upon mRNA seq analysis

**Additional file 3: Figure S3.** Distribution of degraded fragments of *GhMYB2* mRNA. *GhMYB109* was used as the negative control for the miR828 targeting. GhphasiRNA1 was predicted to be phasiRNA deriving genes in the cotton genome to serve as positive control. *UBL1-Gh* was used as the negative control for non-miRNA targeting loci. PhasiRNA may play a role in mRNA degradation at its origin loci, as shown in the anther degradome data. They may also play roles in other target sites, as shown in the hypocotyl degradome distribution and heat map

**Additional file 4: Figure S4.** Functional enrichment analysis of the down-regulated gene group from Fig. 5 panel b in both hypocotyl and anther tissues

**Additional file 5: Dataset 1**

**Additional file 6: Dataset 2**

**Additional file 7: Table S1.** phasiRNA read number in small RNA libraries from two phasing frames on GhMYB2

**Additional file 8: Table S2.** The mapping statistics for the small RNA libraries and degradome libraries used in this study

**Additional file 9: Table S3.** The miR173 and miR390 predicted target in cotton genome

**Additional file 10: Table S4.**

**Additional file 11: Table S5.** Gene accession and primer

**Additional file 12: Table S6**. The sequences of miRNA precursors

## Data Availability

The datasets used and analyzed during the current study available at The National Center for Biotechnology Information, Gene Expression Omnibus (NCBI GEO, https://www.ncbi.nlm.nih.gov/geo/) with accession number of PRJNA293171, GSE43531 (anther, small RNA and degradome) and GSE41132 (hypocotyl and embryogenic calli, small RNA and degradome). The plant materials are available from the corresponding author on reasonable request.

## Abbreviations

phasiRNA: Phased small interfering RNA
RLM-RACE: RNA ligase-mediated rapid amplification of cDNA ends
phasiRNA: Trans-acting small interfering RNA
EC: Embryogenic calli
PF: Phasing frame
DPA: Days post anthesis
DEGs: Differentially expressed genes
