# Supplementary figures and images for: Role of phasiRNAs from two distinct phasing frames of GhMYB2 loci in cis- gene regulation in the cotton genome
Source: BMC Plant Biol. 2020 May 15;20:219. doi: 10.1186/s12870-020-02430-3 (PMC7227086; doi:10.1186/s12870-020-02430-3)

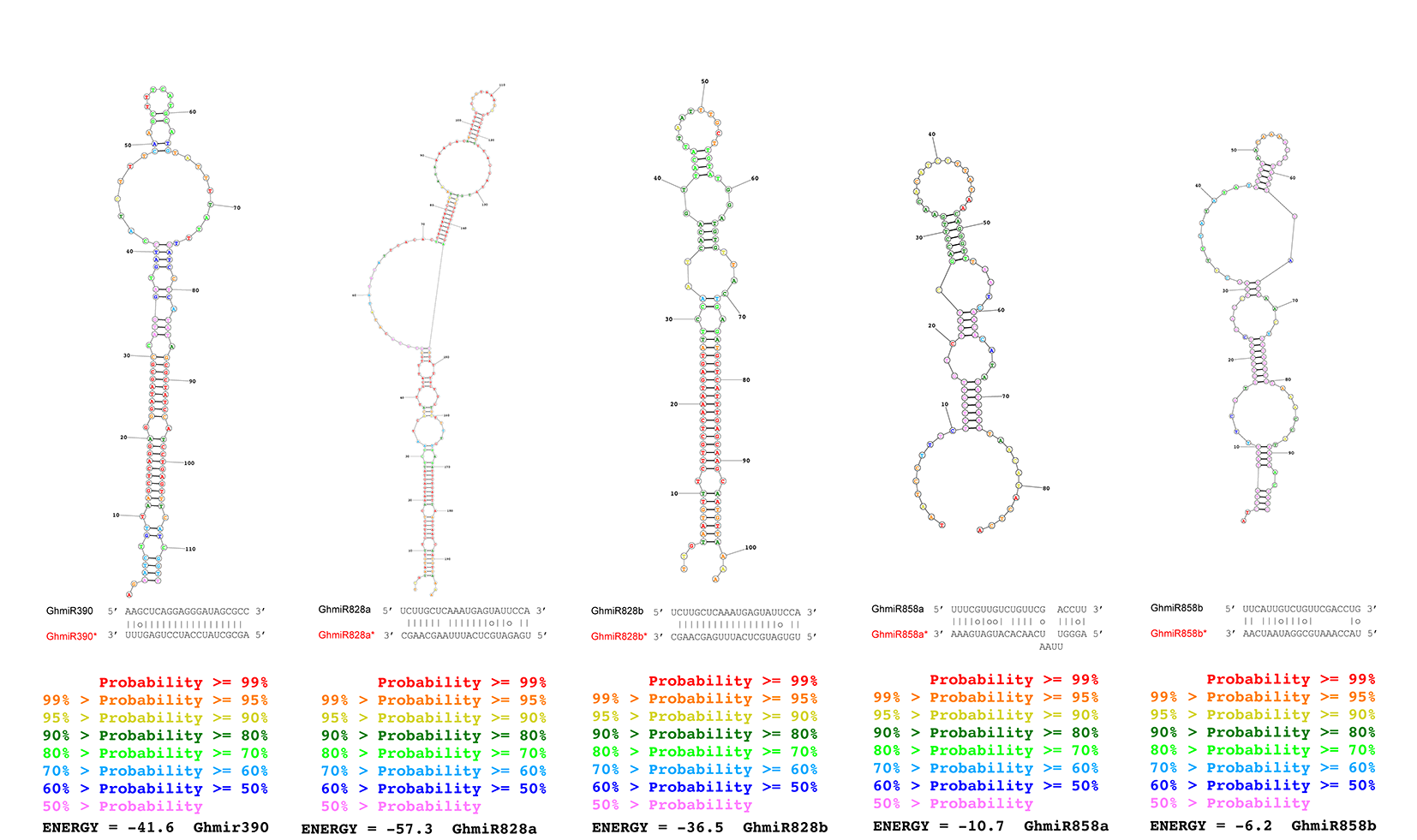

Supplement: Supplementary file 1 — Additional file 1: Figure S1. The secondary structure of miR828 and miR858 precursors [file 12870_2020_2430_MOESM1_ESM.tif]

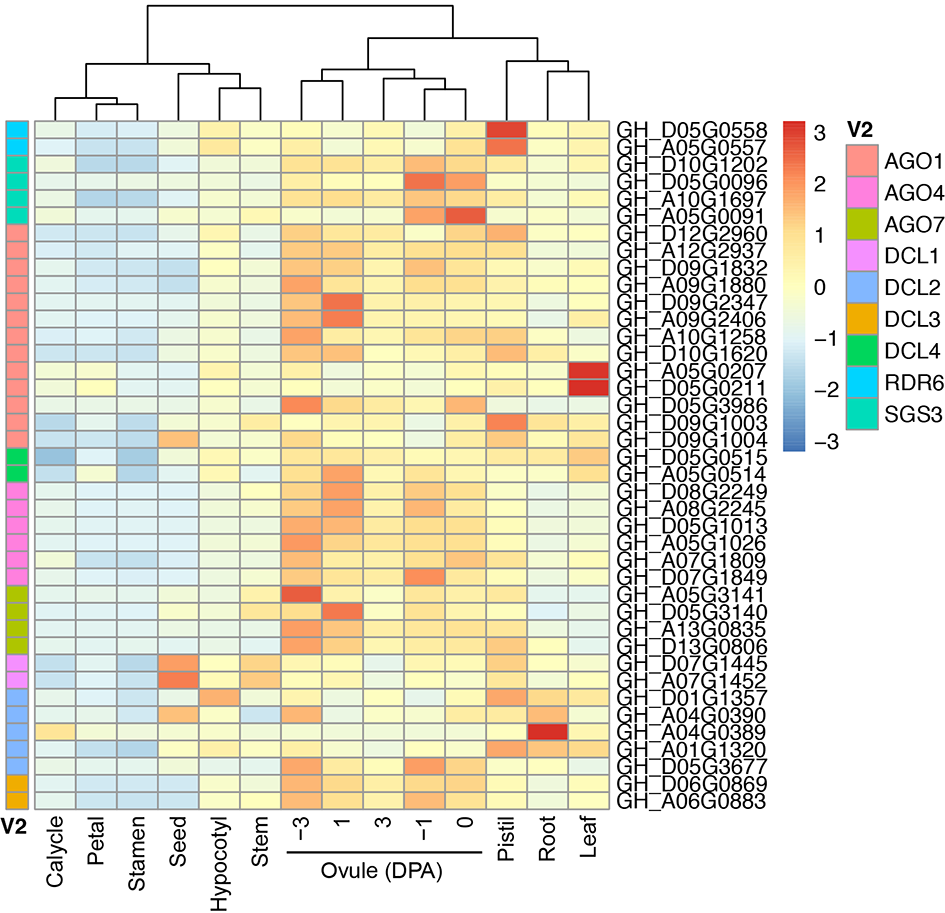

Supplement: Supplementary file 2 — Additional file 2: Figure S2. The heatmap of mRNA activity showing the AGO, DCL, RDR6, SGS3 homologs in upland cotton genome based upon mRNA seq analysis [file 12870_2020_2430_MOESM2_ESM.tif]

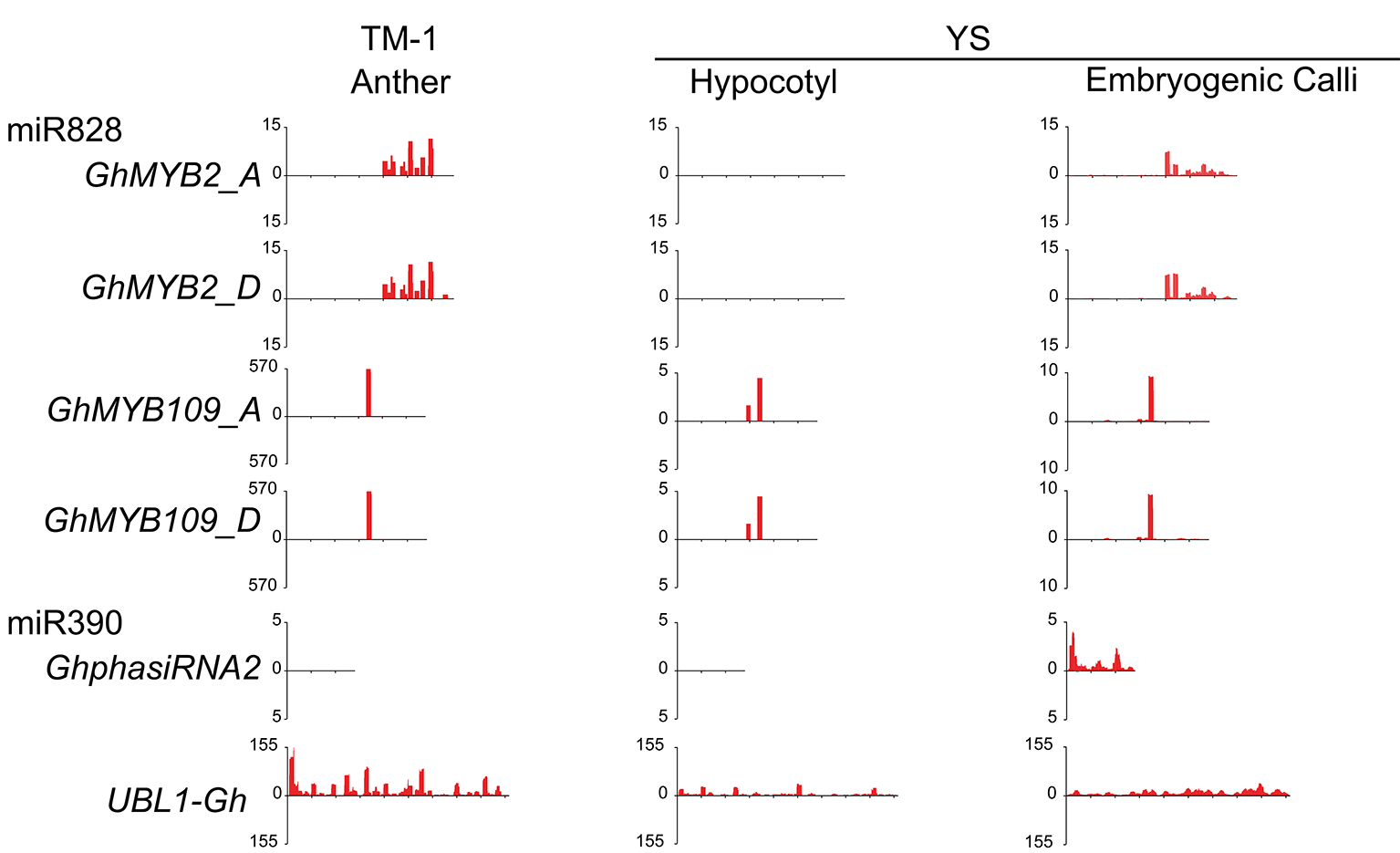

Supplement: Supplementary file 3 — Additional file 3: Figure S3. Distribution of degraded fragments of GhMYB2 mRNA. GhMYB109 was used as the negative control for the miR828 targeting. GhphasiRNA1 was predicted to be phasiRNA deriving genes in the cotton genome to serve as positive control. UBL1-Gh was used as the negative control for non-miRNA targeting loci. PhasiRNA may play a role in mRNA degradation at its origin loci, as shown in the anther degradome data. They may also play roles in other target sites, as shown in the hypocotyl degradome distribution and heat map [file 12870_2020_2430_MOESM3_ESM.tif]

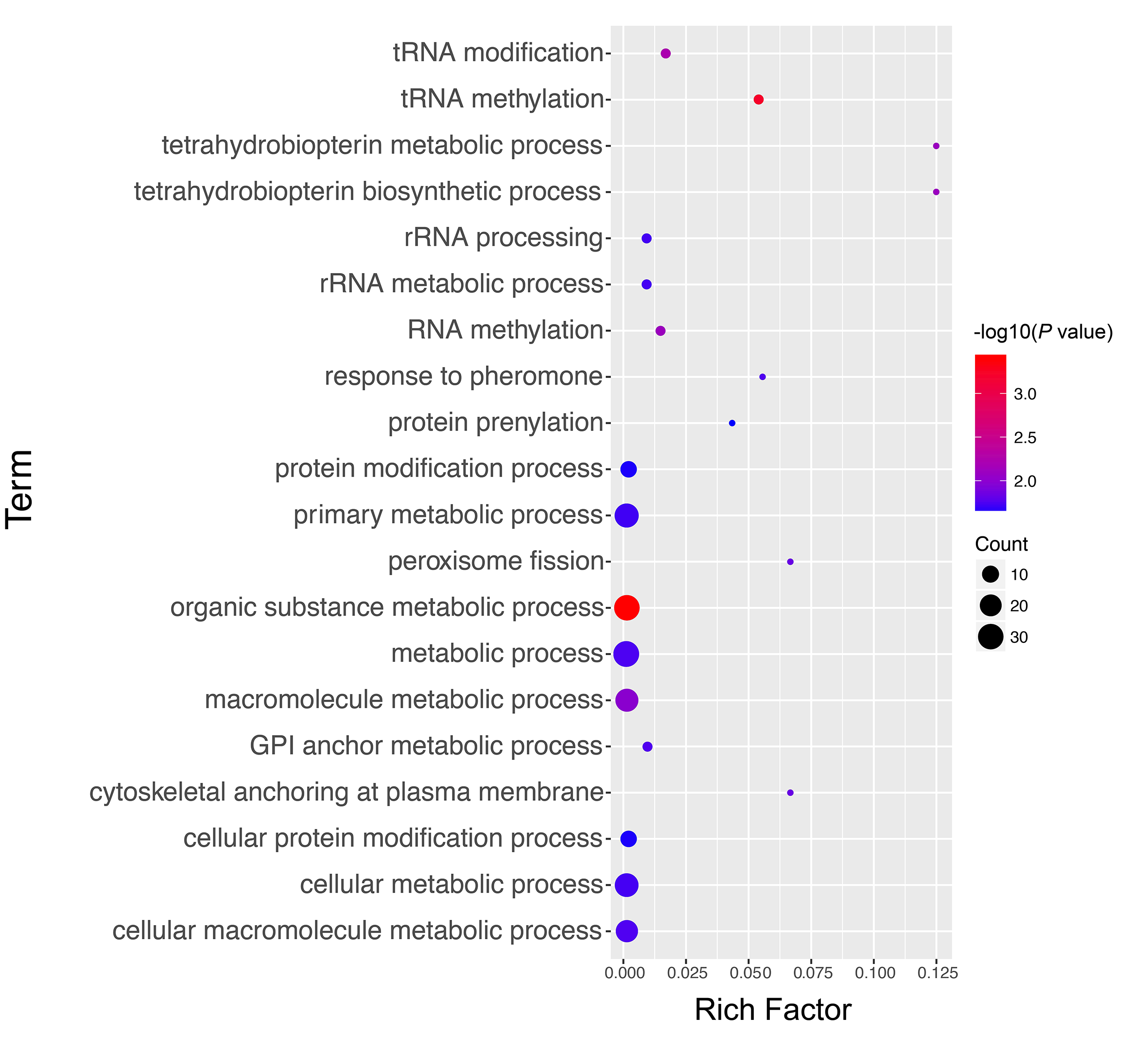

Supplement: Supplementary file 4 — Additional file 4: Figure S4. Functional enrichment analysis of the down-regulated gene group from Fig. 5 panel b in both hypocotyl and anther tissues [file 12870_2020_2430_MOESM4_ESM.tif]
